# Enhanced Passive Bat Rabies Surveillance in Indigenous Bat Species from Germany - A Retrospective Study

**DOI:** 10.1371/journal.pntd.0002835

**Published:** 2014-05-01

**Authors:** Juliane Schatz, Conrad Martin Freuling, Ernst Auer, Hooman Goharriz, Christine Harbusch, Nicholas Johnson, Ingrid Kaipf, Thomas Christoph Mettenleiter, Kristin Mühldorfer, Ralf-Udo Mühle, Bernd Ohlendorf, Bärbel Pott-Dörfer, Julia Prüger, Hanan Sheikh Ali, Dagmar Stiefel, Jens Teubner, Rainer Günter Ulrich, Gudrun Wibbelt, Thomas Müller

**Affiliations:** 1 Friedrich-Loeffler-Institut, Federal Research Institute for Animal Health, WHO Collaborating Centre for Rabies Surveillance and Research, Greifswald - Insel Riems, Germany; 2 Arbeitskreis Fledermäuse Bodensee-Oberschwaben, Naturschutzbund Deutschland e.V., Überlingen, Germany; 3 Wildlife Zoonoses and Vector Borne Diseases Research Group, Animal Health and Veterinary Laboratories Agency (AHVLA), Weybridge, Surrey, United Kingdom; 4 Naturschutzbund Saarland e.V., Arbeitsgemeinschaft Fledermausschutz, Perl-Kesslingen, Germany; 5 Eberhard Karls Universität Tübingen, Tübingen, Germany; 6 Freie Universität Berlin, Berlin, Germany; 7 University of Potsdam, Department of Animal Ecology, Potsdam, Germany; 8 Biosphärenreservat Karstlandschaft Südharz, Landesreferenzstelle für Fledermausschutz Sachsen-Anhalt, Roβla, Germany; 9 Niedersächsischer Landesbetrieb für Wasserwirtschaft, Küsten- und Naturschutz, Hannover, Germany; 10 Interessengemeinschaft für Fledermausschutz und -forschung in Thüringen e.V., Schweina, Germany; 11 Friedrich-Loeffler-Institut, Institute for Novel and Emerging Infectious Diseases, Greifswald - Insel Riems, Germany; 12 Staatliche Vogelschutzwarte für Hessen, Rheinland-Pfalz und Saarland, Frankfurt am Main, Germany; 13 Landesamt für Umwelt, Gesundheit und Verbraucherschutz Land Brandenburg, Naturschutzstation Zippelsförde, Zippelsförde, Germany; 14 Leibniz-Institute for Zoo- und Wildlife Research, Berlin, Germany; The Global Alliance for Rabies Control, United States of America

## Abstract

In Germany, rabies in bats is a notifiable zoonotic disease, which is caused by European bat lyssaviruses type 1 and 2 (EBLV-1 and 2), and the recently discovered new lyssavirus species Bokeloh bat lyssavirus (BBLV). As the understanding of bat rabies in insectivorous bat species is limited, in addition to routine bat rabies diagnosis, an enhanced passive surveillance study, i.e. the retrospective investigation of dead bats that had not been tested for rabies, was initiated in 1998 to study the distribution, abundance and epidemiology of lyssavirus infections in bats from Germany. A total number of 5478 individuals representing 21 bat species within two families were included in this study. The Noctule bat (*Nyctalus noctula*) and the Common pipistrelle (*Pipistrellus pipistrellus*) represented the most specimens submitted. Of all investigated bats, 1.17% tested positive for lyssaviruses using the fluorescent antibody test (FAT). The vast majority of positive cases was identified as EBLV-1, predominately associated with the Serotine bat (*Eptesicus serotinus*). However, rabies cases in other species, i.e. Nathusius' pipistrelle bat (*Pipistrellus nathusii*), *P. pipistrellus* and Brown long-eared bat (*Plecotus auritus*) were also characterized as EBLV-1. In contrast, EBLV-2 was isolated from three Daubenton's bats (*Myotis daubentonii*). These three cases contribute significantly to the understanding of EBLV-2 infections in Germany as only one case had been reported prior to this study. This enhanced passive surveillance indicated that besides known reservoir species, further bat species are affected by lyssavirus infections. Given the increasing diversity of lyssaviruses and bats as reservoir host species worldwide, lyssavirus positive specimens, i.e. both bat and virus need to be confirmed by molecular techniques.

## Author Summary

According to the World Health Organization rabies is considered both a neglected zoonotic and a tropical disease. The causative agents are lyssaviruses which have their primary reservoir in bats. Although bat rabies is notifiable in Germany, the number of submitted bats during routine surveillance is rarely representative of the natural bat population. Therefore, the aim of this study was to include dead bats from various sources for enhanced bat rabies surveillance. The results show that a considerable number of additional bat rabies cases can be detected, thus improving the knowledge on the frequency, geographical distribution and reservoir-association of bat lyssavirus infections in Germany. The overall proportion of positives was lower than during routine surveillance in Germany. While the majority of cases were found in the Serotine bat and characterized as European bat lyssavirus type 1 (EBLV-1), three of the four EBLV-2 infections detected in Germany were found in *Myotis daubentonii* during this study.

## Introduction

Lyssaviruses are non-segmented negative-strand RNA viruses of the order Mononegavirales, family Rhabdoviridae and causative agents of rabies in bats and other mammals as well as in humans [1]. While rabies in dogs and other carnivores has been known since antiquity, the first evidence of rabies in haematophagous and insectivorous bats was reported from the Americas in the first half of the 20th century [2]. Since 1954, bat rabies cases have also been reported from other continents. Antigenic and genetic analyses revealed the diversity of different lyssavirus species, and to date, besides classical rabies virus (RABV), thirteen additional lyssaviruses have been discovered, mostly in bats [3]. Beyond Europe, Lagos bat virus (LBV), Mokola virus (MOKV), Duvenhage virus (DUVV), Shimoni bat virus (SHBV), and Ikoma lyssavirus (IKOV) were found in Africa. In Asian bat species, Aravan virus (ARAV), Khujand virus (KHUV), and Irkut virus (IRKV) were isolated. With the exception of MOKV and IKOV, all of those viruses were detected in bats [3]. In Australia, which has a long history of freedom from classical rabies, Australian bat lyssavirus (ABLV) is found in insectivorous and pteropid bats [4].

In Europe, bat rabies is also caused by several lyssavirus species. Between 1977 and 2012, a total of 1039 bat rabies cases were reported from European countries (http://www.who-rabies-bulletin.org). The majority was characterized as European bat lyssavirus type 1 (EBLV-1) isolated from *Eptesicus* bat species (*E. serotinus, E. isabellinus*) [5]. Genetically, EBLV-1 can be divided in two subtypes, EBLV-1a and 1b [6], [7]. While the EBLV-1a subtype is predominantly found in Central and Eastern Europe (France, The Netherlands, Denmark, Germany and Poland), EBLV-1b has been reported from Spain, France, Southern Germany, and central Poland [8]–[11].

European bat lyssavirus type 2 (EBLV-2) has been isolated from Daubenton's bats in the UK, Switzerland, Finland and Germany, and from Pond bats (*M. dasycneme*) in The Netherlands [12]. As of today, three Natterer's bats (*Myotis nattereri*) infected with the novel Bokeloh bat lyssavirus (BBLV) have been found in Germany and France [13]–[15]. A single detection of the West Caucasian bat virus (WCBV) in a Schreiber's bent-winged bat (*Miniopterus schreibersii*) has been reported from Western Caucasus Mountains [16]. Interestingly, specific RNA from a putative new lyssavirus named Lleida bat lyssavirus (LLEBV) was detected in brain material from the same bat species collected in Spain [17].

The public health relevance of bat rabies in general is highlighted by the fact that most of the bat associated lyssaviruses have caused human rabies [18]. In Europe, both EBLV-1 and EBLV-2 were responsible for four confirmed human casualties [19]. Also, sporadic spill-over infections of EBLV-1 to terrestrial mammals have been reported, i.e. in sheep in Denmark, two cats in France and a stone marten (*Martes foina*) in Germany [20]–[22].

Because of the zoonotic character of bat lyssaviruses knowledge about distribution, abundance and epidemiology is important to estimate and subsequently reduce the public health risk posed by bat rabies. Guidelines for the surveillance of bat lyssaviruses in Europe were established by the European research consortium Med-Vet-Net [23] and supported by EUROBATS [24]. The investigation of sick or dead bats for lyssavirus antigen in brain samples (passive surveillance) and testing of oro-pharyngeal swab samples and serum samples from free-living indigenous bats (active surveillance) for the presence of viral RNA or virus neutralizing antibodies, respectively, were recommended. However, the levels of active and passive bat rabies surveillance in Europe are still very heterogeneous despite previous recommendations [5]. Based on published data, active surveillance provides only limited information and cannot replace passive bat rabies surveillance [25].

Comprehensive passive bat rabies surveillance was conducted in The Netherlands [26], the United Kingdom [27], France [28] and Germany [10]. With the exception of Germany, passive surveillance in these countries is realized by only one or two cooperating departments investigating all bats submitted from the whole country. In contrast, rabies diagnosis in Germany is the responsibility of the sixteen federal states [10]. Dead or diseased bats with symptoms suggestive of rabies, particularly after contact with humans (bites and scratches) have to be submitted and tested for lyssavirus infection in the regional veterinary laboratories.

While cases of this notifiable disease in carnivores and bats were reported to the National Reference Laboratory for Rabies at the WHO Collaborating Centre for Rabies Surveillance and Research (FLI Riems, Germany), the number of bats tested negative was only sporadically submitted. Furthermore, the identification of bats to species level is generally missing as in some other European countries [5]. Therefore, routine bat rabies surveillance in Germany has relied on limited and opportunistic sampling which may not be representative of the true epidemiological situation [10].

To overcome these limitations and to obtain further information on the epidemiology of bat rabies in Germany an enhanced passive retrospective surveillance study was started at FLI in 1998. In this study, the focus was on dead bats excluded from routine diagnostic testing. This included bats obtained from (private) collections from different parts of Germany. Each sample was identified to species level, partly by molecular tools and tested for lyssavirus infection. Here, we present the data from this study and compare it with published data from routine diagnostic screening.

## Material and Methods

### Ethics statement

Dead bats were submitted under the prevailing laws of the respective federal states and following EUROBATS guidelines [24]. Because this study was in the frame of a surveillance programme conducted by the national reference laboratory for rabies no further permits were necessary.

### Sample collection

Starting in 1998, on a federal state level bat conservationists, as well as various institutions and authorities (e.g. Nature and Biodiversity Conservation Union (NABU, Germany), Museum of Natural Science, wildlife care centers) were requested and encouraged to submit dead bats for rabies diagnosis irrespective of the circumstances of acquisition. Archived or newly acquired bats were submitted from all 16 German federal states by local bat biologists during long-time monitoring or routine inspection of maternity roosts, wintering grounds or were killed by cats, wind turbines or unintentional removal of roosts. All bats were stored frozen prior to submission in a chilled state.

### Bat identification

Usually, bat carcasses were submitted with additional information, e.g. geographical origin, date found, sex, age and species identification. Bats without species information were determined to genus or even to species level using external morphological features [29]. Bat carcasses which were degraded or damaged and those suspected to represent cryptic bat species (e.g. *Myotis mystacinus*, *M. brandtii* and *M. alcathoe*) were identified by a mitochondrial *cytochrome b* (*cyt b*) gene specific PCR [30] if not restricted by museum specific preservation requirements. For this purpose, wing membrane samples were collected and stored separately in Eppendorf tubes at −80°C until analysis. For DNA preparation a small piece (1.0×1.0 mm) of each sample was lysed overnight (56°C, 400 rpm) using 3 µl proteinase K (10 mg/ml) and 300 µl lysis buffer (50 mM KCL, 10 mM TRIS-HCL (pH 9.0), 0.45% nonidet NP 40 and 0.45% Tween 20). After centrifugation (1 min, 13000 rpm) the supernatant was stored at −20°C. For PCR amplification two primer pairs (CytB Uni fw: 5′-CATCMTGATGAAAYTTYGG-3′ and CytB Uni rev: 5′-ACTGGYTGDCCBCCRATTCA-3′ [30]; HG for: 5′-CACTACACATCAGAYAC-3′ and HG rev: 5′-AAGGCGAAGAATCGRGT-3′) were used to obtain fragments of about 950 bp and 400 bp, respectively. The latter primer mix was developed based on reference material submitted to the AHVLA to identify all bat species indigenous to the UK (data not shown).

The PCR reaction mix (total volume of 25 µl) consisted of RNase-free water (17.65 µl), 25 mM of each dNTP (0.5 µl), 50 mM MgCl2 (0.75 µl), 10 pmol/µl of each primer (0.5 µl), 10x PCR RxN Buffer (2.5 µl), (0.1 µl) Platinum-Taq DNA Polymerase (Invitrogen, Darmstadt, Germany) and 2.5 µl template DNA. The amplification was performed with the following temperature profile: 3 min at 94°C (initial denaturation), followed by 50 cycles of 30 s at 94°C (denaturation), 30 s at 47°C (annealing), 1 min at 72°C (elongation) and a final extension at 72°C for 10 min. Amplification of the expected products was confirmed in a 1% agarose gel stained with ethidium bromide or SYBR safe DNA gel stain. PCR products were then purified (NucleoSpin Gel and PCR Clean-up kit, Macherey-Nagel, Düren, Germany) and sequenced using BigDye Terminator v1.1 Cycle Sequencing Kit (Applied Biosystems/Life Technologies, Carlsbad, CA, USA). The *cytochrome b* sequences were compared with published sequences of European bat species (GenBank) using the Basic Local Alignment Search Tool (BLAST, http://blast.ncbi.nlm.nih.gov/Blast.cgi) and the species determination was finalized by identification of the species of the highest nucleotide sequence similarity (≥90%).

### Fluorescent antibody test (FAT)

Rabies diagnosis was performed on bat brain samples which were removed either by opening of cranium or, in case of natural scientific collections, by puncture of *foramen occipitale magnum* using a 26-gauge needle. Lyssavirus antigen was detected by standard fluorescent antibody test (FAT) using commercially available polyclonal fluorescein isothiocyanate (FITC)-labelled anti-rabies conjugates (Behring, Marburg; SIFIN, Berlin, Germany) following standard protocols [31]. Additional tests included virus isolation in cell culture, reverse-transcription quantitative real-time polymerase chain reaction (RT-qPCR) and sequencing following RT-PCR was performed to confirm positive FAT results.

### Rabies Tissue Culture Infection Test (RTCIT)

For virus isolation, FAT positive or inconclusive bat brain samples were homogenized in a volume of 1000 µl sterile minimum essential medium (MEM-10, with 2% Streptomycin). The resulting brain suspensions (500 µl) were subjected to the RTCIT [32], using a mouse neuroblastoma cell line (MNA 42/13, No. 411, cell culture collection for veterinary medicine, FLI). Infected cells were incubated for three days at 37°C and 5% CO_2_ and then tested using FAT. A result was confirmed negative after the third consecutive cell passage.

### Detection of lyssavirus RNA using Polymerase Chain Reaction (PCR)

RNA was extracted from 200 µl brain suspension or RTCIT supernatant using TRIzol Reagent (Invitrogen, Darmstadt, Germany)/peqGOLD TriFast (peqlab Biotechnologie GmbH, Erlangen, Germany) method. The RNA pellet was re-suspended in a volume of 20 µl bidistilled water. Samples were analysed for the presence of viral RNA using quantitative real-time PCR (RT-qPCR) specific for EBLV-1/-2 as described [25]. In cases of inconclusive FAT results a conventional panlyssavirus RT-PCR was additionally performed [33].

### Sequence and phylogenetic analysis

All EBLV-isolates were further characterized by sequence analysis [34]. RNA was subjected to one-step RT-PCR using primers JW12 and JW6 E [33] followed by sequencing. Briefly, after amplification, PCR-products were run in a 1% agarose gel stained with ethidium bromide, excised and purified essentially as for the molecular bat species identification. Sequences were manually checked for quality, trimmed to the first 400 bp using SeqMan (Lasergene, DNASTAR, Madison, WI, USA)) and submitted to NCBI GenBank (Table S1). Sequence alignment and subsequent phylogenetic analysis was performed using MEGA 5. Further representatives of EBLV-1 and 2 were derived from GenBank for comparison (Table S2).

## Results

**Figure 1:**
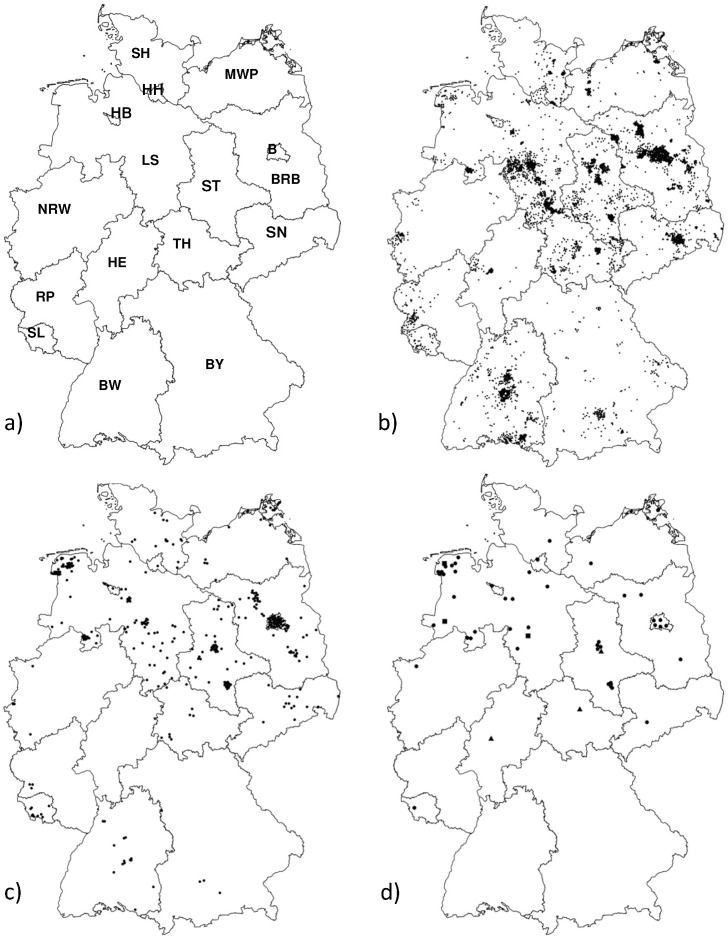
Map showing federal states of Germany (a) and geographical origin of all bat specimens coming from Schleswig-Holstein (SH, N = 362); Bremen (HB, N = 4), Hamburg (HH, N = 10), Mecklenburg-Western Pomerania (MWP, N = 131), Lower Saxony (LS, N = 1252), Berlin (B, N = 484), Brandenburg (BRB, N = 644), Saxony-Anhalt (ST, N = 692), Saxony (SN, N = 247), North Rhine Westphalia (NRW, N = 76), Hesse (HE, N = 89), Thuringia (TH, N = 296), Rhineland-Palatinate (RP, N = 108), Saarland (SL, N = 53), Baden-Wuerttemberg (BW, N = 736), Bavaria (BY, N = 252) (b) and of *E. serotinus* (c) collected in the study described here, and of the bat rabies cases (dot (N = 46): *E. serotinus*, triangle (N = 3): *M. daubentonii*, square (N = 3): *P. pipistrellus, P. nathusii and Pl. auritus* (d).

From 1998 to June 2013 a total of 5478 bats from all German federal states (N = 16, Figure 1b) were investigated. The annual number of submissions to FLI of obtained specimens varied between 30 and 1200 individuals. The bats encompassed specimens from the entire study period and before, with the oldest sample originating from 1981.

Among all samples, 21 out of the 23 indigenous bat species in Germany were included (Table 1), although the proportion of bat species differed per federal state. The majority of bat samples originated from Lower Saxony (N = 1252), followed by Baden-Wuerttemberg (N = 736) and Saxony-Anhalt (N = 692). In contrast, in three and two of the remaining federal states the sample size was less than 90 and 15, respectively.

**Table 1:** Number of bat samples per species investigated using FAT, RTCIT, RT-qPCR and RT-PCR.

			Bat brain samples tested for lyssaviruses using:							
	Number of bats		FAT				RTCIT	RT-qPCR (positive)		RT-PCR
Bat species	submitted	sequenced (*cyt b*)	not analysable	negative	positive	inconclusive	positive	EBLV-1	EBLV-2	positive
*Barbastella barbastellus*	15		1	14						
*Eptesicus nilssonii*	46		5	41						
*Eptesicus serotinus*	386	3	17	313	50	6	48	49	-	49
*Myotis bechsteinii*	17	3	3	14						
*Myotis brandtii*	73	7	14	59						
*Myotis dasycneme*	8		1	6		1	-	-	-	-
*Myotis daubentonii*	185	6	25	146	3	11	3	-	3	3
*Myotis emarginatus*	1			1						
*Myotis myotis*	171	1	38	131		2	-	-	-	-
*Myotis mystacinus*	207	3	31	173		3	-	-	-	-
*Myotis nattereri*	176	8	17	155		4	-	-	-	-
*Nyctalus leisleri*	72	2	7	65						
*Nyctalus noctula*	1329	4	109	1209		11	-	-	-	-
*Pipistrellus kuhlii*	9			9						
*Pipistrellus nathusii*	278	10	50	226	1	1	1	1	-	1
*Pipistrellus pipistrellus*	1694	59	277	1411	1	5	1	1	-	1
*Pipistrellus pygmaeus*	28	4	6	22						
*Plecotus auritus*	341	6	40	298	1	2	1	1	-	1
*Plecotus austriacus*	87	2	16	71						
*Rhinolophus hipposideros*	1			1						
*Vespertilio murinus*	143	1	16	127						
unknown	211		45	165		1	-	-	-	-
**total**	**5478**	**119**	**718**	**4657**	**56**	**47**	**54**	**52**	**3**	**55**

With the exception of a single carcass of the Lesser horseshoe bat (*Rhinolophus hipposideros*), all other species investigated belonged to the family Vespertilionidae. Among those, the most frequently tested bat species were the Common pipistrelle and Noctule bat followed by Serotine bat and Brown long-eared bat (Table 1). A total of 330 bats could not be identified to species level using external morphological criteria. *Cyt b* sequences were obtained from 119 bats, representing 15 different species (Table 1). The sequence similarity ranged between 92% and 100% when compared to publicly available sequences. Wing membrane samples from the remaining 211 individuals from natural scientific collections were not available.

Most positive specimens were found in bats from Lower Saxony (N = 27), Saxony-Anhalt (N = 10) and Berlin (N = 5) (Figure 1d). Bat rabies was detected in animals from additional 10 German federal states although only sporadically (1–3 cases). No lyssavirus infection was found in bats originating from Rhineland-Palatinate (N = 108), Baden-Wuerttemberg (N = 736) and Bavaria (N = 252) (Figure 1b–d).

**Figure 2:**
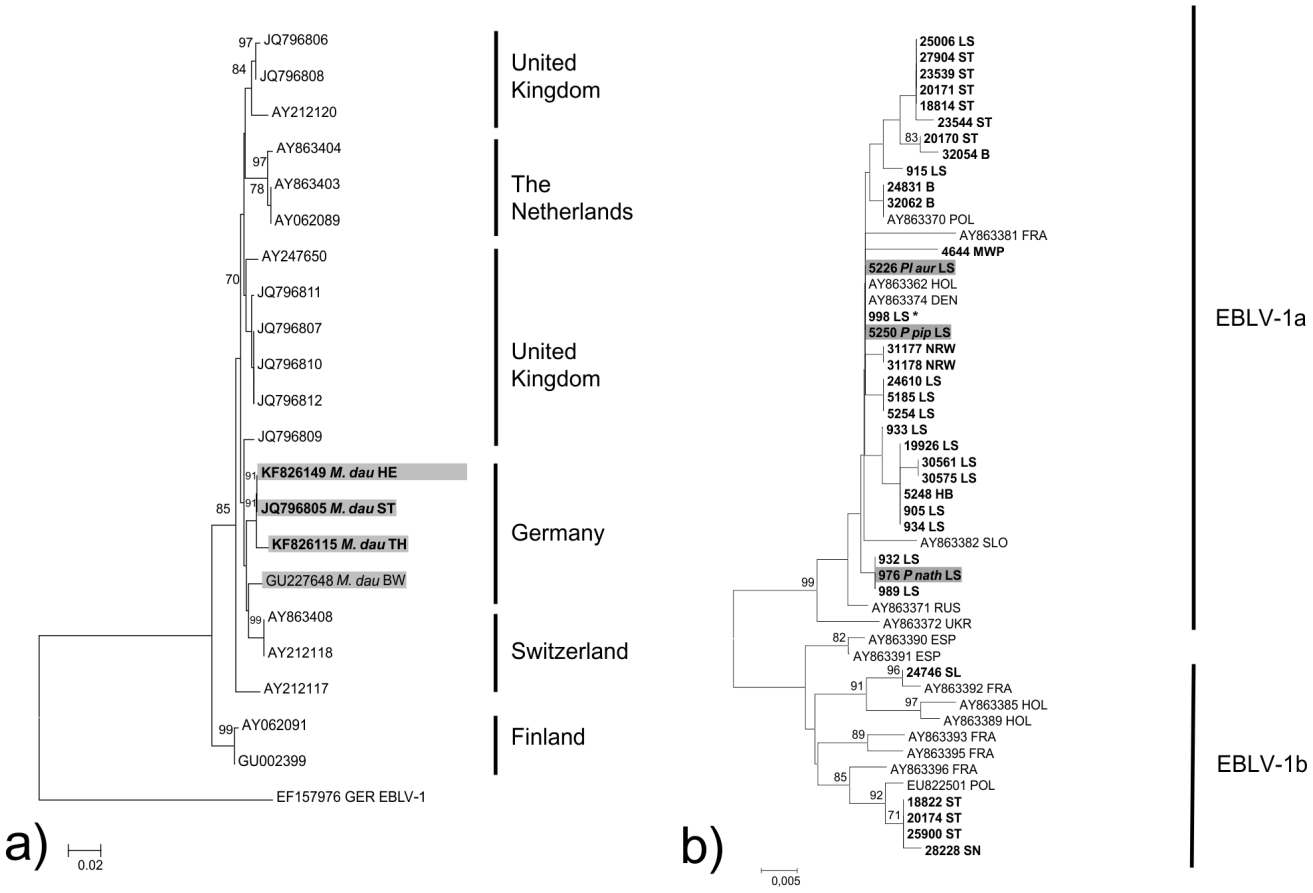
Evolutionary relationships of EBLV-1 (a) and EBLV-2 strains (b) with a focus on 400 nucleotides long N-gene sequences (nt positions 1–400, numbering according to EF157976) derived from this study (boldface). The Neighbor-Joining method (p-distance, 1000 pseudoreplicates) as implemented in MEGA 5 was used. Sequence number 998 LS represents the identical sequences 959, 5300, 5304, 7471, 7467, 11647, 15730, 16902, 16908, 18720, 21836, 24525, 24529, 24832, 25495, 31054.

Except for a single Serotine bat for which sufficient brain material was not available, lyssaviruses were successfully isolated and sequenced from 54 and 55 bats, respectively, which had been tested FAT-positive (Table 1). The presence of EBLVs was confirmed in five different bat species (*E. serotinus, P. pipistrellus, P. nathusii, Pl.auritus* and *M. daubentonii*). The majority of viruses were identified as EBLV-1, predominately isolated from *E. serotinus* (N = 48). Single lyssavirus infections in other species were also characterized as EBLV-1 (Table 1). The phylogenetic analysis of the N gene derived sequences identified the two lineages of EBLV-1, i.e. five out of the 52 available sequences were characterized as EBLV-1b found in Serotine bats originating from Saarland (N = 1), Saxony-Anhalt (N = 3) and Saxony (N = 1) (Figure 2a). Some clustering was observed for EBLV-1a isolates from the same or from neighbouring federal states, with occasional exceptions. The nucleotide sequence divergence within the EBLV-1a group was <1%.

Of 160 Daubenton's bats tested, three (1.88%) individuals were rabies positive, and EBLV-2 was isolated in each case (Table 1, Figure 2b). Those infected bats were submitted from Saxony-Anhalt [35], Thuringia and Hesse. Overall, in 47 smears from different bat species investigated using the FAT small fluorescing structures indicative for lyssavirus antigen was found, but the infection could not be confirmed by other methods, e.g. RTCIT, EBLV-1/-2 specific RT-qPCR and conventional RT-PCR. Furthermore, 13.1% (N = 718) of all submitted bats could not be investigated because the carcasses were mummified or organs had autolysed (Table 1).

**Figure 3:**
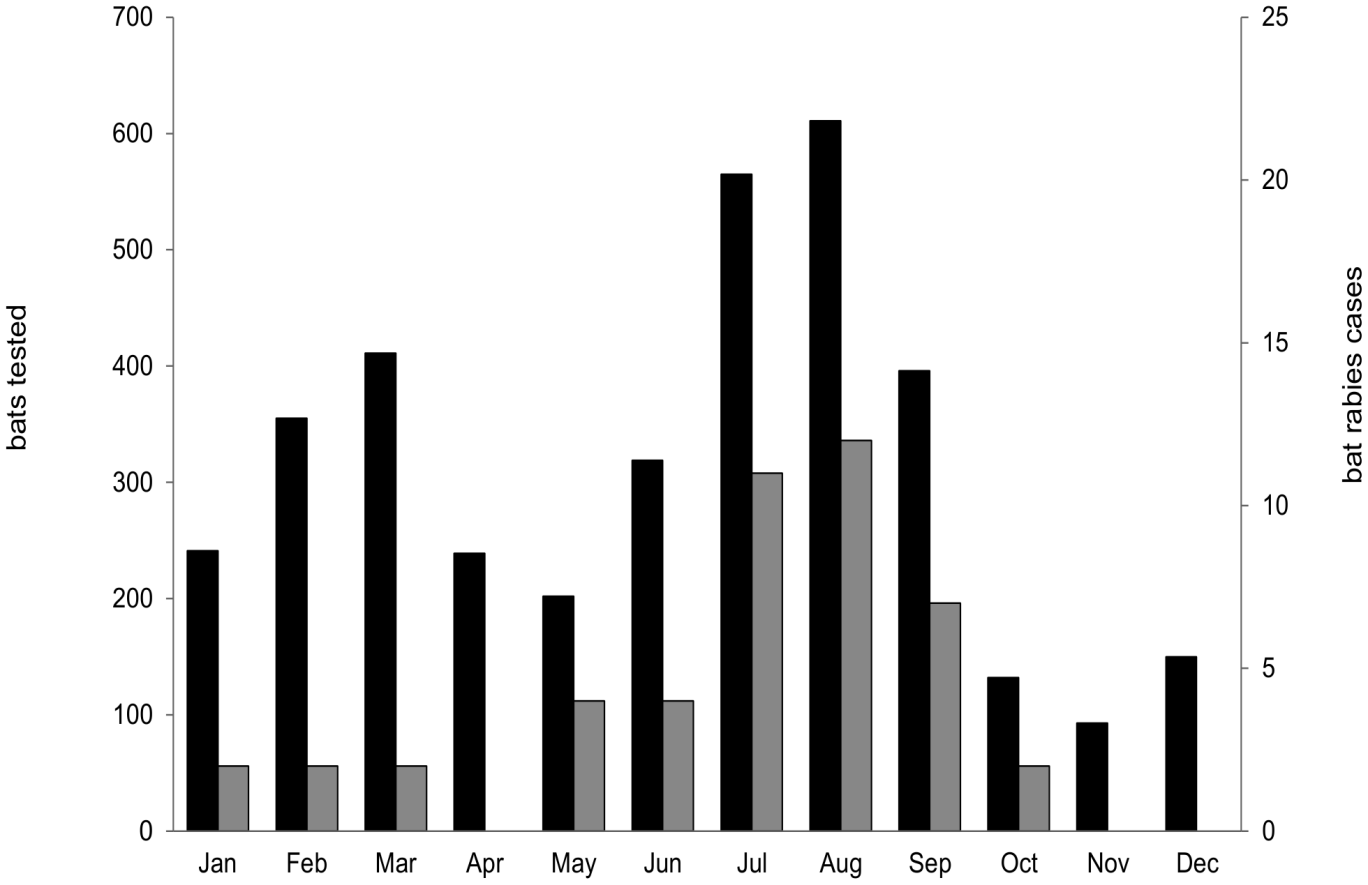
Number of bat specimens tested (N = 3714, black) and rabies cases (N = 46, grey) per month during 1998 until June 2013.

Of all animals tested by FAT and with a reference to a date (month, N = 3714) the peak of bat finds were in July, August and September, with a second peak in February and March (Figure 3). Of those, the percentage of bats tested EBLV-positive was highest in July (N = 11, 1.95%) and August (N = 12, 1.96%). Altogether, 50 Serotine bats tested rabies positive by FAT, of which 18 were males and 11 females. Four of the positive cases were juvenile animals whereas the remaining animals were sub-adults or adults.

## Discussion

In Germany, routine bat rabies surveillance has been reliant on limited and opportunistic sampling as a result of international, national and federal state specific legislation that often restricts the handling, submission and even testing of bats [10]. Because the current knowledge about distribution, abundance and epidemiology of bat lyssaviruses is rather fragmentary, we initiated long-term enhanced passive bat rabies surveillance in Germany. When this study was started in 1998 it was a prerequisite to collect samples through extensive lobbying, education and awareness training of local bat biologists at a federal state level to encourage submissions of bat carcasses. Only with the 2006 Agreement on the Conservation of Populations of Bats in Europe by EUROBATS [24], which for the first time established a basis for legitimized bat rabies surveillance in Europe, the collection, submission and testing of indigenous dead bats was enabled. Within 15 years of this retrospective study (1998-June 2013) with more than 5000 bats, a six fold higher number of indigenous bats was tested for lyssavirus infections, compared to the number of bats examined during 50 years of routine surveillance in Germany [10]. We could therefore demonstrate that enhanced passive surveillance can generate an increase in submissions. Comparable studies were also conducted in The Netherlands (1984–2003, N = 3873), the UK (1987–2004, N = 4883), France (1989–2004, N = 934), and Switzerland (1976–2009, N = 837) [5], although we were able to sample more bats in a shorter period of time. Together with routine surveillance (data not shown) the passive bat rabies surveillance in Germany appears to be more intense than in most other European countries [5].

In this enhanced passive surveillance bats were submitted which had rarely been included in routine surveillance because they have a low likelihood of human contact (e.g. bats from caves, forests etc.) or bats from the countryside. Samples from e.g. private bat collections were included that had been stored in freezers for up to 25 years. Despite this long period of storage it was still possible to isolate lyssavirus from those brain tissues.

In this retrospective study, 56 additional bat rabies cases were detected that otherwise would have been missed. Together with the number of positive cases from routine surveillance (1977–2012, N = 243) (www.who-rabies-bulletin.org) Germany is one of the countries in Europe with the highest number of reported bat rabies cases. Evidently, the high level of surveillance contributes to this fact. However, the influence of other factors such as abundance of reservoir species and virus prevalence needs to be studied further.

To date three different bat lyssaviruses have been reported from Germany. While the presence of EBLV-1 has been known for a considerable period of time [10], EBLV-2 [33] and BBLV [13] were first isolated in 2007 and 2010, respectively, during routine bat rabies surveillance. In this retrospective study we confirm both the circulation of EBLV-1 and EBLV-2 in Germany.

However, samples included in this study comprise almost all bat species indigenous in Germany. Depending on the geographical distribution, population density and their habitat use the number of submissions varied considerably per bat species. Similar to the UK, the Netherlands and Switzerland the Common pipistrelle was the most frequently submitted bat species [26], [27], [36]. In fact, this synanthropic species is also one of the most abundant bat species in Europe. Although rabies in this species had been reported before [37], [38], in this study we confirmed an EBLV-1 infection for the first time by RTCIT, RT-qPCR and sequencing. EBLV-1 infections in species other than the Serotine bat (*E. serotinus*) was also found in a single Nathusius' pipistrelle bat and a Brown long-eared bat. All those viruses were identified as EBLV-1 and showed geographical clustering thus indicating that they resemble spill-over infections from infected Serotine bats [10].

In contrast to North America, where RABV is found in most bat species with distinct lineages [39], this pattern was not found for EBLV-1 in European bats.

A total of 49 Serotine bats tested EBLV-1 positive confirming that this bat species is the main reservoir for EBLV-1. Surprisingly, despite testing of individuals of this bat species originating from various regions in Germany, the majority of EBLV-1 cases was found in Serotine bats from the northwest of Germany, supporting previous studies [10]. Although the Serotine bat is abundant all over Germany, the density is higher in the northern lowlands of Germany [40], suggesting that the intraspecies transmission rate is higher so that more cases are detected, both in routine as well as in enhanced surveillance. While the situation appears similar in the Netherlands [26], the distribution of positive EBLV-1 cases in France differs insofar as those infections were detected in many parts of the country irrespective of the altitude [28].

The majority of lyssaviruses were characterized as EBLV-1a. Similar to previous studies from the Netherlands and Germany [10], [26], [41], EBLV-1a sequences showed a very high level of identity. However, genetic clusters appear to be linked to defined geographic regions (Figure 2a). In the past, EBLV-1b had been sporadically detected in the German federal state of Saarland close to the French border [10], [41]. Surprisingly, we could confirm the presence of the EBLV-1b subtype also in central and eastern parts of Germany. Genetically, those isolates are more closely related to an EBLV-1b isolate from Poland than with the other Saarland isolate (Figure 2a). Since Serotine bats generally do not migrate, the sporadic occurrence of EBLV-1b variants in Germany and Poland remains puzzling. However, our results could reflect a recent eastward spread of EBLV-1b although this needs further investigation.

Whilst during routine surveillance in Germany only a single Daubenton's bat was found to be infected with EBLV-2 [33], we report three additional cases. Because Daubenton's bats are associated with forest habitat, detection of grounded bats by the public is limited. This is reflected by the low number of Daubenton's bats tested for lyssavirus infections in other European studies [5]. In our study a total of 160 Daubenton's bats were tested for bat lyssaviruses resulting in an estimated prevalence of 1.88% for EBLV-2. This is comparable to estimates for Switzerland (4.6%) and the UK (3.6%). The pond bat was associated with EBLV-2 infection in the Netherlands [26]. We were only able to test seven individuals of this species, and thus cannot properly assess whether this species serves as a true reservoir host or represent a spill-over infection from Daubenton's bat.

Generally, irrespective of the low number of tested bats, the prevalence of EBLV-2 in Daubenton's bats seems to be lower than EBLV-1 in Serotine bats. In our study we found EBLV-1 in 13.28% of all tested Serotine bats, whilst in Spain (*E. isabellinus*) and in the Netherlands this proportion of positives was 20% and 21%, respectively [26], [42].

Overall, 47 brain smears of 11 bat species, including the reservoir bat species *E. serotinus*, *M. daubentonii* and *M. nattereri*, showed a particulate staining pattern morphologically similar to anti-rabies staining in FAT. This could be regarded as typical but lyssavirus infection could not be confirmed with further tests e.g. RT-qPCR, RT-PCR or RTCIT. False positive results have been shown to occur in diagnosis of classical rabies, but at a very low percentage [43]. Degradation of samples and microbial contamination may lead to certain cross-reactions with the anti-rabies conjugates. Furthermore, cross-reactions with other viral encephalites, such as West Nile and Powassan flavivirus infection cannot be excluded [44]. Besides in reservoir species, a large proportion of these unspecific results were found in the Noctule bat. Previously, bat rabies cases had been reported sporadically in this species [45], although cases were not confirmed by virus isolation and/or sequence analysis. Further investigations are needed to establish the cause of this observation.

While in this retrospective study none of the 159 Natterer's bats tested positive for BBLV, during an enhanced passive bat rabies study performed in the German federal state Bavaria BBLV was isolated from a single Natterer's bat [15]. Although with more than 20000 captures per year this is the most handled bat of all reservoir bats species (*M. nattereri, E. serotinus, M. daubentonii, M. dasycneme*) known to exist in Germany [D. Brockmann, Bat Marking Centre Dresden, Germany, pers. communication], this bat species is clearly underrepresented in passive surveillance due to its sylvatic mode of life making it difficult to find large numbers of dead bats of this species.

As stated before, EBLV-1 infections were not only detected in *E. serotinus* but also in three other indigenous bat species. In contrast to this study, during routine surveillance only about half of FAT positive bats were determined to species level where the majority of cases in Serotine bats were identified [10]. Thus, it cannot be excluded that more bat species are affected by lyssavirus infections. This demonstrates the importance of species identification for epidemiological evaluation as previously shown for the UK [46]. In cases where morphological species identification was not possible due to either the quality of the specimens (e.g. damaged, degraded) or the absence of morphological criteria, (i.e. cryptic bat species), samples were genetically characterized. By this we were able to characterize more than 100 bat specimens which helped to complete the dataset. Given the increasing diversity of lyssaviruses and reservoir bat species, lyssavirus positive specimens, i.e. both bat and virus need to be confirmed by molecular techniques. For example, the bat species originally described as associated with KHUV and ARAV may be incorrect [4]. Similarly, SHBV isolated from *Hipposideros vittatus* in Kenya, was initially described as *Hipposideros commersonii* [47]. Furthermore, genetic information on hosts may allow for a comparison with the viral evolution as shown for North America [39] and Eastern Europe [48].

Based on the experience gained in this project, we propose that enhanced passive surveillance for bat rabies should be continued to complement routine diagnosis. Thus, it is a prerequisite to collect dead bats as “fresh” as possible and freeze them as soon as possible. To this end, a close cooperation with all stakeholders involved in bat handling, monitoring and research is essential. Those dead bats should eventually be transferred to a central point where rabies diagnosis can be performed. In parallel, bats involved in human contact have to be tested by the responsible regional veterinary laboratories, to allow for prompt veterinary and human public health response. All bats need to be identified to species level by morphological and/or molecular techniques. Finally, it is of eminent importance that all data are combined into a comprehensive evaluation.

Research activities, particularly surveillance efforts to gain insights into the epidemiology of bat lyssaviruses, can be regarded as a true bat conservation effort, since a greater understanding of this zoonosis can help to reduce unjustified fear and misconceptions.

### Conclusions

With enhanced passive surveillance 56 additional bat rabies cases were detected also in federal states where rabies in bats had not been found previously. Considering the large number of animals tested the prevalence was lower than in routine surveillance and likely represents the true level of lyssavirus infections in indigenous bats in Germany. Although the vast majority of cases were found in the known reservoir species *Eptesicus serotinus*, spill-over cases were also observed. In conclusion, all bat species need to be sampled and identified, and, since some bat species are still underrepresented, the enhanced surveillance should be maintained.

## Supporting Information

Table S1 Details of EBLV isolates from Germany.
(DOCX)

Table S2 Details of additional EBLV N-gene sequences included in the phylogenetic analysis.
(DOCX)
