# Supplementary material for: Enhanced Passive Bat Rabies Surveillance in Indigenous Bat Species from Germany - A Retrospective Study
Source: PLoS Negl Trop Dis. 2014 May 1;8(5):e2835. doi: 10.1371/journal.pntd.0002835 (PMC4006713; doi:10.1371/journal.pntd.0002835)
Supplement: Table S1 — Details of EBLV isolates from Germany. (DOCX) [file pntd.0002835.s001.docx]

**Supplementary table 1**.

| **ID. No.** | **Genotype** | **Bat species** | **Year** | **Location** | **Federal state** | **GenBank accesion no.** | **Reference** |
| --- | --- | --- | --- | --- | --- | --- | --- |
| 905 | EBLV-1a | *E. serotinus* | n. i. | Zippelsförde | Brandenburg | KF826138 | this study |
| 915 | EBLV-1a | *E. serotinus* | 1996 | Osnabrück | Lower Saxony | KF826139 | this study; Freuling et al., 2012 |
| 932 | EBLV-1a | *E. serotinus* | 1991 | Breddenburg | Lower Saxony | KF826140 | this study; Müller et al., 2007 |
| 933 | EBLV-1a | *E. serotinus* | 1993 | Oldendorf | Lower Saxony | KF826141 | this study; Müller et al., 2007 |
| 934 | EBLV-1a | *E. serotinus* | 1997 | Osnabrück | Lower Saxony | KF826142 | this study; Müller et al., 2007 |
| 959 | EBLV-1a | *E. serotinus* | 1988 | Aurich | Lower Saxony | KF826143 | this study; Müller et al., 2007 |
| 976 | EBLV-1a | *P. nathusii* | 1992 | Marienhafe | Lower Saxony | KF826144 | this study; Freuling et al., 2012 |
| 989 | EBLV-1a | *E. serotinus* | n. i. | Aurich | Lower Saxony | KF826145 | this study; Müller et al., 2007 |
| 992 | EBLV-1a | *E. serotinus* | 1998 | Moordorf | Lower Saxony | KF826146 | this study; Müller et al., 2007 |
| 998 | EBLV-1a | *E. serotinus* | 1997 | Emden | Lower Saxony | KF826147 | this study; Müller et al., 2007 |
| 4644 | EBLV-1a | *E. serotinus* | 1997 | Plate | M.-W.-Pomerania | KF826128 | this study; Freuling et al., 2012 |
| 5185 | EBLV-1a | *E. serotinus* | 2000 | Hitzhausen | Lower Saxony | KF826129 | this study; Freuling et al., 2012 |
| 5226 | EBLV-1a | *Pl. auritus* | 1996 | Lingen | Lower Saxony | KF826130 | this study; Müller et al., 2007 |
| 5248 | EBLV-1a | *E. serotinus* | n. i. | Bremen | Bremen | KF826131 | this study; Freuling et al., 2012 |
| 5250 | EBLV-1a | *P. pipistrellus* | 1994 | Hannover | Lower Saxony | KF826132 | this study; Müller et al., 2007 |
| 5254 | EBLV-1a | *E. serotinus* | n. i. | Osterode | Lower Saxony | KF826133 | this study; Müller et al., 2007 |
| 5300 | EBLV-1a | *E. serotinus* | 1999 | Emden | Lower Saxony | KF826134 | this study; Müller et al., 2007 |
| 5304 | EBLV-1a | *E. serotinus* | 1999 | Emden | Lower Saxony | KF826135 | this study; Müller et al., 2007 |
| 7467 | EBLV-1a | *E. serotinus* | 2000 | Emden | Lower Saxony | KF826136 | this study; Müller et al., 2007 |
| 7471 | EBLV-1a | *E. serotinus* | 2000 | Emden | Lower Saxony | KF826137 | this study; Müller et al., 2007 |
| 11647 | EBLV-1a | *E. serotinus* | 2005 | Kyritz | Brandenburg | KF826097 | this study; Freuling et al., 2012 |
| 15730 | EBLV-1a | *E. serotinus* | 2005 | Lübbenau | Brandenburg | KF826096 | this study; Freuling et al., 2012 |
| 16902 | EBLV-1a | *E. serotinus* | n. i. | Aurich | Lower Saxony | KF826098 | this study |
| 16908 | EBLV-1a | *E. serotinus* | 2004 | Stedesdorf | Lower Saxony | KF826099 | this study |
| 18720 | EBLV-1a | *E. serotinus* | 2002 | Thedinghausen | Lower Saxony | KF826100 | this study |
| 18814 | EBLV-1a | *E. serotinus* | 2007 | Magdeburg | Saxony-Anhalt | KF826102 | this study |
| 18822 | EBLV-1b | *E. serotinus* | 2005 | Magdeburg | Saxony-Anhalt | KF826103 | this study |
| 18856 | EBLV-2 | *M. daubentonii* | 2006 | Magdeburg | Saxony-Anhalt | JQ796805 | Freuling et al. 2012 |
| 19926 | EBLV-1a | *E. serotinus* | 2008 | Nordhorn | Lower Saxony | KF826104 | this study |
| 20170 | EBLV-1a | *E. serotinus* | 2008 | Halle/Saale | Saxony-Anhalt | KF826105 | this study |
| 20171 | EBLV-1a | *E. serotinus* | 2008 | Halle/Saale | Saxony-Anhalt | KF826106 | this study |
| 20174 | EBLV-1b | *E. serotinus* | 2008 | Magdeburg | Saxony-Anhalt | KF826107 | this study |
| 21836 | EBLV-1a | *E. serotinus* | 2010 | Bergen | M.-W.-Pomerania | KF826101 | this study |
| 23539 | EBLV-1a | *E. serotinus* | 2007 | Halle/Saale | Saxony-Anhalt | KF826108 | this study |
| 23544 | EBLV-1a | *E. serotinus* | 2009 | Halle/Saale | Saxony-Anhalt | KF826109 | this study |
| 24525 | EBLV-1a | *E. serotinus* | 2006 | Moisburg | Lower Saxony | KF826110 | this study |
| 24529 | EBLV-1a | *E. serotinus* | n. i. | Verden | Lower Saxony | KF826111 | this study |
| 24610 | EBLV-1a | *E. serotinus* | 2000 | n. i. | Lower Saxony | KF826112 | this study |
| 24746 | EBLV-1b | *E. serotinus* | 2009 | Dillingen/ Saar | Saarland | KF826113 | this study |
| 24831 | EBLV-1a | *E. serotinus* | n. i. | Berlin | Berlin | KF826114 | this study |
| 24832 | EBLV-1a | *E. serotinus* | n. i. | Berlin | Berlin | KF826116 | this study |
| 25006 | EBLV-1a | *E. serotinus* | 2003 | Langenhagen | Lower Saxony | KF826117 | this study |
| 25495 | EBLV-1a | *E. serotinus* | 2010 | Hamburg | Hamburg | KF826118 | this study |
| 25538 | EBLV-2 | *M. daubentonii* | 2006 | Schwansee | Thuringia | KF826115 | this study |
| 25900 | EBLV-1b | *E. serotinus* | 2011 | Magdeburg | Saxony-Anhalt | KF826119 | this study |
| 27904 | EBLV-1a | *E. serotinus* | 2011 | Halle/Saale | Saxony-Anhalt | KF826120 | this study |
| 28228 | EBLV-1a | *E. serotinus* | 2012 | Chemnitz | Saxony | KF826121 | this study |
| 30561 | EBLV-1a | *E. serotinus* | 2010 | Rehburg-Loccum | Lower Saxony | KF826148 | this study |
| 30575 | EBLV-1a | *E. serotinus* | 2010 | Hameln | Lower Saxony | KF826122 | this study |
| 31054 | EBLV-1a | *E. serotinus* | 2010 | Bad Segeberg | Schleswig-Holstein | KF826123 | this study |
| 31177 | EBLV-1a | *E. serotinus* | 2011 | n. i. | North Rhine-Westphalia | KF826124 | this study |
| 31178 | EBLV-1a | *E. serotinus* | 2011 | Hamminkeln | North Rhine-Westphalia | KF826125 | this study |
| 31452 | EBLV-2 | *M. daubentonii* | 2013 | Gießen | Hesse | KF826149 | this study |
| 32054 | EBLV-1a | *E. serotinus* | 2012 | Berlin | Berlin | KF826126 | this study |
| 32062 | EBLV-1a | *E. serotinus* | 2012 | Berlin | Berlin | KF826127 | this study |
