# Supplementary material for: Enhanced Passive Bat Rabies Surveillance in Indigenous Bat Species from Germany - A Retrospective Study
Source: PLoS Negl Trop Dis. 2014 May 1;8(5):e2835. doi: 10.1371/journal.pntd.0002835 (PMC4006713; doi:10.1371/journal.pntd.0002835)
Supplement: Table S2 — Details of additional EBLV N-gene sequences included in the phylogenetic analysis. (DOCX) [file pntd.0002835.s002.docx]

**Supplementary Table 2**.

| GenBank accesion no. | Virus species | Bat species | Year | Location | Country | Reference |
| --- | --- | --- | --- | --- | --- | --- |
| AY062089 | EBLV-2 | *M. dasycneme* | 1989 | Andijk | Netherlands | [7] |
| AY062091 | EBLV-2 | Human | 1985 | Helsinki | Finland | [49] |
| AY212117 | EBLV-2 | *M. daubentonii* | 1992 | Plaffeien | Switzerland | [36] |
| AY212118 | EBLV-2 | *M. daubentonii* | 1993 | Versoix | Switzerland | [7] |
| AY212120 | EBLV-2 | *M. daubentonii* | 2002 | Lancashire | England | [50] |
| AY863404 | EBLV-2 | *M. dasycneme* | 1993 | Roden | Netherlands | [7] |
| AY863408 | EBLV-2 | *M. daubentonii* | 2002 | Geneva | Switzerland | [7] |
| EF157977 | EBLV-2 | Human | 2002 | Angus, Scotland | UK | [51] |
| EU293114 | EBLV-2 | *M. dasycneme* | 1987 | Wommels | Netherlands | [7] |
| GU002399 | EBLV-2 | *M. daubentonii* | 2009 | Turku | Finland | [52] |
| JQ796806 | EBLV-2 | *M. daubentonii* | 2009 | West Lothian, Scotland | United Kingdom | [53] |
| JQ796807 | EBLV-2 | *M. daubentonii* | 2004 | Surrey, England | UK | [54] |
| JQ796808 | EBLV-2 | *M. daubentonii* | 2003 | Lancashire, England | UK | [55] |
| JQ796809 | EBLV-2 | *M. daubentonii* | 2006 | Oxfordshire, England | UK | [56] |
| JQ796810 | EBLV-2 | *M. daubentonii* | 2007 | Shropshire, England | UK | [57] |
| JQ796811 | EBLV-2 | *M. daubentonii* | 2008 | Surrey, England | UK | [58] |
| JQ796812 | EBLV-2 | *M. daubentonii* | 2008 | Shropshire, England | UK | [59] |
| EF157976 | EBLV-1 | *E. serotinus* | 1968 | Hamburg | Germany | [60] |
| AY863381 | EBLV-1 | *E. serotinus* | 2003 | Angers | France | [7] |
| AY863382 | EBLV-1 | *E. serotinus* | 2001 | Presov | Slovakia | [7] |
| AY863385 | EBLV-1 | *E. serotinus* | 1993 | Apeldoorn | Netherlands | [7] |
| AY863389 | EBLV-1 | *E. serotinus* | 1997 | Apeldoorn | Netherlands | [7] |
| AY863390 | EBLV-1 | *E. isabellinus* | 1987 | Granada | Spain | [7] |
| AY863391 | EBLV-1 | *E. isabellinus* | 1994 | Granada | Spain | [7] |
| AY863392 | EBLV-1 | *E. serotinus* | 1989 | Briey | France | [7] |
| AY863393 | EBLV-1 | *E. serotinus* | 1989 | Bainville-sur-Madon | France | [7] |
| AY863395 | EBLV-1 | *E. serotinus* | 1995 | Morlaix Finiste`re | France | [7] |
| AY863396 | EBLV-1 | *E. serotinus* | 2000 | Premilhat | France | [7] |
| AY863372 | EBLV-1 | *V. murinus* | 1987 | Volyn region | Ukraine | [7] |
| AY863371 | EBLV-1 | Human | 1985 | Belgorod | Russia | [7] |
| AY863370 | EBLV-1 | *E. serotinus* | 1994 | Dziekanow | Poland | [7] |
| EU822501 | EBLV-1 | *E. serotinus* |  |  | Poland | unpublished |
| AY863362 | EBLV-1 | *E. serotinus* | 1987 | Bellingwolde | Netherlands | [7] |
| AY863374 | EBLV-1 | *E. serotinus* | 1987 | Christiansfeld | Denmark | [7] |

UK : United Kingdom
